# Characterization of Integrin Molecular Tension of Human Breast Cancer Cells on Anisotropic Nanopatterns

**DOI:** 10.3389/fmolb.2022.825970

**Published:** 2022-06-09

**Authors:** Kyung Ah Kim, Srivithya Vellampatti, Byoung Choul Kim

**Affiliations:** Department of Nano-Bioengineering, Incheon National University, Incheon, South Korea

**Keywords:** mechanobiology, integrin tension, tension gauge tether, molecular force sensor, cancer, anisotropic nanopattern

## Abstract

Physical interactions between cells and micro/nanometer-sized architecture presented in an extracellular matrix (ECM) environment significantly influence cell adhesion and morphology, often facilitating the incidence of diseases, such as cancer invasion and metastasis. Sensing and responding to the topographical cues are deeply associated with a physical interplay between integrins, ligands, and mechanical force transmission, ultimately determining diverse cell behavior. Thus, how the tension applied to the integrin-ligand bonds controls cells’ response to the topographical cues needs to be elucidated through quantitative analysis. Here, in this brief research report, we reported a novel platform, termed “topo-tension gauge tether (TGT),” to visualize single-molecule force applied to the integrin-ligand on the aligned anisotropic nanopatterns. Using the topo-TGT assay, first, topography-induced adhesion and morphology of cancerous and normal cells were compared with the pre-defined peak integrin tension. Next, spatial integrin tensions underneath cells were identified using reconstructed integrin tension maps. As a result, we characterized each cell’s capability to comply with nanotopographies and the magnitude of the spatial integrin tension. Altogether, the quantitative information on integrin tension will be a valuable basis for understanding the biophysical mechanisms underlying the force balance influencing adhesion to the topographical cues.

## Introduction

Topographical cues play an important role in cell behavior, including adhesion, proliferation, differentiation, migration, and tissue formation and function (Kim et al., 2012). Cells change their orientation and migrate along anisotropically arrayed structures, and this phenomenon is referred to as “contact guidance (CG)” (Dickinson et al., 1994; Tranquillo, 1999; Ray et al., 2017). The physical interactions between cells and micro/nanometer-sized architecture presented in their microenvironment facilitate the regulation of physiological and pathophysiological processes (Tranquillo, 1999; Czirok, 2013; Reig et al., 2014). Recent cancer studies have reported that sensing of the topographical features and consequent alteration in cell morphology and function are highly implicated in cancer invasion and migration (Provenzano et al., 2006; Provenzano et al., 2008; Ray et al., 2017; Shin et al., 2017; Tabdanov et al., 2018). For example, compared with normal cells or benign tumors, malignant breast cancer cells migrate with clusters of linear collagen fibers and metastasize away from the primary tumor site to other parts of the body (Wang et al., 2002; Provenzano et al., 2006). Also, metastatic cancer cells are polarized and elongated, following the direction of the aligned nanopatterns by changing the cytoskeleton structure, ultimately resulting in directed migration (Ray et al., 2017). Thus, mechanisms underlying how cancer cells sense the anisotropic patterns and change their behavior and functions should be thoroughly investigated to increase our understanding of cancer progression and find therapeutic targets for cancer treatment.

Cell adhesion and migration are deeply associated with a physical interplay between integrins, which are mechano-sensitive membrane proteins consisting of *α* and *β* subunits and extracellular matrices (ECMs), such as collagen and fibronectin (Hynes, 1992; Gallant et al., 2005; Arnaout et al., 2007). Upon integrin engagement with ECM molecules, a series of intracellular signaling pathways are being processed to form focal adhesion (FA) complexes by recruiting adaptor proteins, such as talin and vinculin, and build a physical linkage with intracellular cytoskeletal organization, causing the transmission of mechanical forces experienced or exerted by cells (Gardel et al., 2010; Kanchanawong et al., 2010). The average ligand density and spacing at the nanoscale have a profound effect on the integrin-mediated cell adhesion. It has been reported that integrins successfully cluster to form the FA when the ligand spacing is less than 70 nm, whereas integrins do not cluster when the ligand spacing is larger than 70 nm (Huang et al., 2009; Wang et al., 2013). After adhesion, the force balance between cells and the underlying substrate ultimately determines cell morphology and function (Gardel et al., 2010; Plotnikov et al., 2012). During this process, it was observed that the activation of integrins by mechanical forces increases the binding affinity to the ECM and promotes FA formation and consequent mechano-transduction (Chicurel et al., 1998; Wang and Ha, 2013; Sun et al., 2019). As such, the tension applied to integrins is a critical factor for integrin-mediated mechano-transduction. It is not challenging to predict that integrin tension and CG of cancer cell adhesion are closely connected.

An engineered-DNA–based force probe termed “tension gauge tether (TGT)” and its derivatives have been successful in characterizing the magnitude of tension applied to integrins needed for stable adhesion and spatio-temporal changes in integrin tension during cell’s spreading and migration (Wang and Ha, 2013; Wang et al., 2015; Jo et al., 2018; Zhu et al., 2018; Jo et al., 2021). The TGT is a double-stranded DNA tether conjugated with a ligand for target integrin binding, which is designed to break under strong integrin tension greater than the tension tolerance (*T*_*tol*_) modulated by changing the DNA geometry. Therefore, it enables the quantitative tuning of the magnitude of peak tension experienced by integrins on cells and the measurement of the tension needed to activate integrin-related signaling pathways with single-molecule precision (Wang and Ha, 2013). By integrating a cyclic RGDfK peptide for α_v_β_3_ and α_5_β_1_ integrin binding, the TGT assay has shown that a universal integrin tension of 40 pN is needed for the activation of cell adhesion through a single integrin, and integrin tensions of >54 pN are transmitted through mature FA by the actomyosin activity (Wang et al., 2015). It also indicates that integrin tension above 54 pN is localized at the trailing edge during rapid cell migration (Zhao et al., 2018). Despite the significant advantages for the characterization of integrin tension in cells, conventional TGT assays have been conducted mostly on flat surfaces; therefore, how the CG-inducing topographical cues influence integrin tension upon cancer adhesion remains largely unknown.

Here, in this brief research report, we developed an engineered TGT-coated anisotropic nanopattern providing topographical cues to elucidate a biophysical role of integrin tension in CG-induced adhesion, polarization, and spreading of human breast cancer and normal cells. Using this platform, we first demonstrated how the peak integrin tension applied to the cell lines modulates adhesion, polarization, and elongation on the aligned nanoarchitecture. Next, we investigated the level and distribution of integrin tension exerted by adherent cells on the nanosurface, allowing us to quantify the integrin tension with the nanoscale spatial resolution. Furthermore, we demonstrated how the formation of stress fibers and consequently altered contractility by external biochemical cues influence cells’ compliance to the nanosurface and an alteration in integrin tension. In particular, how metastatic cancer cells were directed toward the CG was scrutinized by changing their cytoskeleton dynamics and force generation. Strikingly, our results revealed that integrin tension required to activate cell adhesion to the nanosurface was not cell type-dependent but universal, while the subcellular level integrin tension exerted by adherent cells on the nanosurface was highly influenced by cell type and their cell response to the nanotopography. This quantitative information on integrin tension will play an essential role in the characterization of the biophysical force balance influencing CG sensing of cancer cells.

## Methods

### Synthesis of Tension Gauge Tethers

A cyclic RGDfK peptide (cRGDfK-NH2, Peptides International, PCI-3696-PI) was conjugated to the 3′ end of the upper DNA strand with a fluorescent dye (Cy3 or Cy5) at its 5′ end using a hetero-bifunctional cross-linker (Sulfo-SMCC, Thermo Fisher Scientific, 22622) as described elsewhere (Wang and Ha, 2013; Jo et al., 2021). The cRGDfK ligand-conjugated DNA strand was purified by gel electrophoresis. Next, the cRGDfK ligand-conjugated DNA strand and its complementary DNA strand containing biotin were annealed *via* hydrogen bonds. The tension tolerance (*T*_*tol*_ = 43 and 54 pN) is determined by the location of the biotin. For the 100 pN TGT, the cRGDfK ligand was directly conjugated to a DNA strand with biotin at the 5′ end, and it was annealed with its complementary DNA strand with a fluorescent dye (Cy3 or Cy5) at the 5′ end. All DNA strands were purchased from Integrated DNA Technologies (IDT, Inc.).

Specific sequence information of each TGT molecule is summarized.

100 pN TGT: upper strand: 5′ -/Cy3 or Cy5/GGC CCG CAG CGA CCA CCC/- 3′
Bottom strand: 3′ -/Biotin/CCG GGC GTC GCT GGT GGG/cRGDfk/- 5′
54 pN TGT: upper strand: 5′ -/Cy3 or Cy5/GGC CCG CAG CGA CCA CCC/cRGDfk/- 3′
Bottom strand: 3′ -/Biotin/CCG GGC GTC GCT GGT GGG- 5′
43 pN TGT: upper strand: 5′ -/5Cy3 or Cy5/GGC CCG CAG CGA CCA CCC/cRGDfk/- 3′
Bottom strand: 3′ -/CCG GGC GTC/Biotin/GCT GGT GGG- 5′

### Preparation of Tension Gauge Tether Surfaces

Glass bottom dishes with/without anisotropic nanopatterns used [the flat surface (SPL Life Sciences, 100350) and the anisotropically nanopatterned surface (Curi Bio, Inc., ANFS-0001-10) or the custom-made surface using E-beam lithography (Jeol, Inc., JBX9300FS)] were prepared. The size of the fabricated nanopatterns was confirmed by scanning electron microscopy imaging (Hitachi, S-4300SE). First of all, to prevent non-specific binding, the glass bottom dishes were coated with 400 μg/ml of biotinylated bovine serum albumin (BSA) (Sigma-Aldrich, A8549) dissolved in the phosphate-buffered saline (PBS) for 2 h at room temperature and rinsed with PBS thrice thoroughly. A measure of 200 μg/ml of neutravidin (Thermo Fisher Scientific, 31000) was loaded onto the surface and incubated for 10 min at room temperature; then, the surface was washed with PBS three times. Then, 1 μl of 1 μM TGT solution was placed at the center of the BSA-coated glass surface and incubated in a humidity chamber for 30 min at room temperature followed by washing with PBS. (Caution! The TGT surfaces should always be remained undried to prevent a messy and enhanced imaging background).

### Preparation of the Fibronectin-Coated Surface

Each glass bottom dish was incubated with a mixture of fibronectin (at the final concentration of 20 μg/ml) and BSA (at the final concentration of 400 μg/ml) in PBS at room temperature. After 2 h of incubation, the surfaces were washed with PBS three times thoroughly. The dishes were filled with PBS to prevent them from drying before use.

### Cell Culture

The human breast cancer cell lines (MDA-MB-231 and MCF7 cells) were cultured in Dulbecco’s modified Eagle’s medium (DMEM, Gibco, 11965-092) supplemented with 10% (v/v) fetal bovine serum (FBS, Gibco, 16000044) and 1% (v/v) penicillin/streptomycin (Gibco, 15140-122). The normal cell line MCF10A was cultured in DMEM/F-12 (Sigma-Aldrich, D8437) supplemented with 5% horse serum (Gibco, 16050122), 20 ng/ml human epidermal growth factor (EGF, Sino Biological, 10605-HNAY), 0.5 μg/ml hydrocortisone (Sigma-Aldrich, H0888), 100 ng/ml cholera toxin (List Labs, #100B), 10 μg/ml insulin (Thermo Fisher Scientific, 12585-014), and 1% penicillin/streptomycin (Gibco, 15140-122). All cells were cultured in the standard incubation condition (5% CO_2_ at 37°C in a humidified environment). Each cell line was harvested from a culture dish and re-suspended in a serum-free medium for the TGT assay. The cells were seeded onto the TGT surfaces at the density of 10^5^ cells/ml. After 2 h of incubation, the cells were fixed with 4% paraformaldehyde for imaging.

### Drug Treatments

Drugs were added to each cell culture dish with 70% cell confluency. For activation of RhoA or inhibition of phosphoinositide 3-kinase (PI3K), a Rho activator (Rho activator II, Cytoskeleton, Inc., CN03) or a PI3K inhibitor (LY294002, Sigma-Aldrich, L9908) in a pure culture medium was added to the culture dish and incubated for 2 h before the TGT assay.

The final concentration of each drug is as follows: Rho Activator II (0.25 μg/ml) and LY294002 (10 μM).

### Immunostaining

Cells were fixed with 4% (v/v) para-formaldehyde (PFA, Samchun Chemicals) in PBS at room temperature for 10 min. After fixation, the culture dishes were washed thrice with PBS, and cells were permeabilized with 0.1% Triton X (Thermo Fisher Scientific, HFH10) for 10 min. Non-specific binding was blocked by 3% BSA solution (Sigma-Aldrich, A9576) after thorough washing. Subsequently, the cells were stained for focal adhesions (FAs) using a focal adhesion staining kit (Sigma-Aldrich, FAK100) and an anti-mouse 488 secondary antibody (BioActs, RSA-1141). The actin cytoskeleton was stained using TRITC-phalloidin, and the nucleus was stained using Hoechst 33342 (Thermo Scientific, 62249).

### Imaging

Differential interference contrast (DIC) images of the fixed cells and fluorescence images of the TGT ruptures and stained proteins were taken by using an epifluorescence microscope (Nikon Ti-2, Nikon Inc.). For cell imaging, ×10 objective (CFI Plan Fluor) and ×60 objective (CFI Plan Apochromat Lambda ×60) were used, and all images were recorded with an electron-multiplying charge-coupled device (EMCCD, Andor iXon Life 888). In addition, a confocal microscope (FV3000, Olympus) and a stage-top live cell imaging chamber (LCI bio, Inc.) were exploited for confocal imaging.

### Image Analysis

Morphological measurements: single cells were selected for the analysis of morphological changes on the nanopatterns. Shape factors including circularity and the projected area of individual cells were measured by ImageJ (NIH). Each shape factor was calculated after drawing the cell boundary manually using the DIC image channel. Circularity varies from 0 for highly irregular shapes to 1 for a perfect circular shape.

### Statistical Analysis

The number of cells (n) and repeats are noted in each figure caption. Mean value with standard error (SE) and other statistical analyses were achieved using GraphPad Prism (GraphPad Software). An unpaired t-test was conducted to estimate significance. The *p*-value was indicated in the figure captures. **** for *p* < 0.0001, *** for *p* < 0.001, ** for *p* < 0.01, and * for *p* < 0.05.

## Results

### Cell Adhesion and Orientation Upon the Anisotropic Nanopatterns are Determined by the Cell Type and Peak Integrin Tension

Three separate human breast epithelial cell lines were used to study how cancer cell adhesion and spreading differ with the topography and magnitude of peak integrin tension: two human breast cancer cell lines, MDA-MB-231 (highly invasive) and MCF7 (noninvasive), and a normal cell line MCF10A as a control group.

**FIGURE 1:**
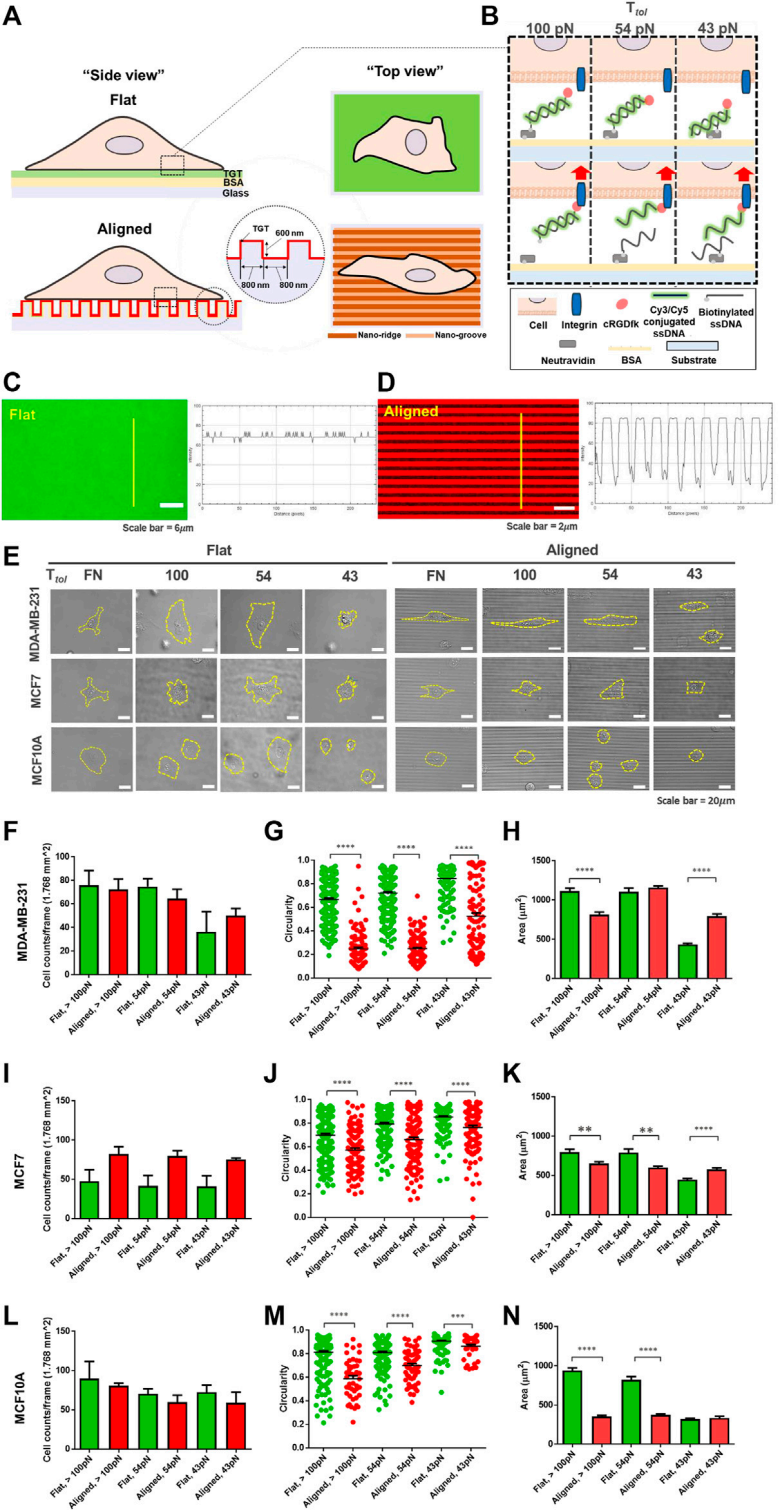
Development of the topo-TGT assay: tension gauge tether (TGT)-coated nanosurface and cell adhesion on the nanosurface. **(A)** Schematic representation of cell adhesion on the single molecule integrin tension probe, tension gauge tether (TGT), and coated surfaces (flat and anisotropic nanopatterns). **(B)** TGT constructs and their working principle. Three different TGT constructs with *T*_*tol*_ of 43, 54, and 100 pN were prepared. **(C, D)** Verification of the TGT coating on each surface. **(C)** Cy3-conjugated TGT molecules were immobilized on the flat surface *via* biotin-neutravidin bonds, and the fluorescent intensity profile was plotted. **(D)** Image of Cy5-conjugated TGT-coated nanosurface. The intensity profile indicates TGT-coating on the aligned nanopatterns. **(E)** Representative images of adherent cells on each surface (incubation time, t = 2 h). Three different human breast epithelial cell lines were used in this study: two human breast cancer cell lines, MDA-MB-231 (highly invasive) and MCF7 (non-invasive), and a normal cell line MCF10A as a control group. Cell adhesion and spreading varied with the cell type and the topographical cue and the magnitude of peak integrin tension. Scale bar, 20 µm. **(F)** Total number of adherent MDA-MB-231 cells per unit area (*n* = 5, 3, 5, 3, 3, and 3), **(G)** circularity (*n* = 232, 105, 240, 138, 206, and 104), and **(H)** projected cell area (*n* = 232, 143, 240, 140, 206, and 104) on each TGT surface. Circularity varies from 0 for highly irregular shapes to 1 for a perfect circular shape. **(I–K)** Total number of adherent MCF7 cells (*n* = 4, 3, 4, 3, 4, and 3), their circularity (*n* = 188, 111, 169, 122, 180, and 116), and projected area (*n* = 188, 139, 169, 123, 180, and 116) on the TGT surfaces. **(L–N)** Total number of adherent MCF10A cells (*n* = 3, 3, 3, 3, 3, and 3), their circularity (*n* = 188, 46, 169, 59, 180, and 41), and projected area (*n* = 188, 83, 169, 95, 180, and 87) on the TGT surfaces. All experiments were repeated at least three times. Sample numbers are denoted. Data are presented as mean ± SE.

First, to differentiate the magnitude of spatial integrin tension at the contact interface, an unpatterned flat surface and an anisotropic nanotopographical surface coated with the TGT, named “topo-TGT,” were prepared (Figures 1A,B). The nanotopographical dimension to provide CG was 800 nm ridges and 800 nm grooves with a height of 600 nm Supplementary Figure S1 because it mimics spacings of fibrils observed in mammary collagens (Provenzano et al., 2006; Ray et al., 2017; Tabdanov et al., 2018). Each surface was passivated with a biotinylated BSA; then, TGT molecules with different tension tolerance (*T*_*tol*_) values of 43, 54, and 100 pN regarding the universal integrin tension of 40 pN for the successful adhesion (Wang and Ha, 2013) were tethered to each surface using a neutravidin-biotin bond; therefore, cell adhesion was entirely mediated *via* the immobilized TGT. For further functionalization, the TGT molecules were conjugated with the cRGDfK ligand targeting the α_v_β_3_ and α_5_β_1_ integrins for cell adhesion and a fluorescent probe (either Cy3 or Cy5 dye) for visualization of the TGT rupture. For 43 and 54 pN TGT, the *T*_*tol*_ was modulated by the location of biotin on the TGT. It was estimated using the de Gennes model and magnetic tweezer experiments regarding the application of the constant force for 2 s (de Gennes, 2001; Hatch et al., 2008). For 100 pN TGT, a single-stranded DNA conjugated with the cRGDfK ligand and biotin in each end is directly tethered to the immobilized neutravidin, causing an enhanced rupture force (greater than 100 pN) (de Odrowaz Piramowicz et al., 2006). Then, the quality of the TGT coating on the flat surface and the nanosurface was analyzed using the fluorescent intensity profile emitted from the tagged Cy3/Cy5 dye with the geometry of the surface (Figures 1C,D). As expected, a uniform intensity profile was observed on the flat surface, whereas periodic intensity changes corresponding to the geometry of the nanopatterns were observed on the nanosurface.

Next, each cell line was loaded on both TGT surfaces and incubated for 2 h. Morphological changes during adhesion in response to the nanotopographical cue and the level of peak integrin tension were examined (Figure 1E). For highly invasive MDA-MB-231 cells, there was no significant difference in the number of cells adhered to each surface per unit area (Figure 1F). However, significantly different circularities were observed because cells on the nanosurface were highly polarized and elongated, following the direction of the patterns compared to cells on the flat surface (Figures 1G,H). This tendency was further strengthened on the nanosurfaces with high *T*_*tol*_ TGTs (54 and 100 pN). Interestingly, in terms of the surface area, for *T*_*tol*_ = 100 pN, cells on the flat surface spread larger than those on the nanosurface because of increased horizontal elongation and limited expansion perpendicular to the axis of the anisotropic nanopatterns. Inversely, for *T*_*tol*_ = 43 pN, cells on the nanosurface were larger due to elongation along the nanopatterns. These results showed that MDA-MB-231 cells enable to change their morphology by combining the surface topography and integrin tension experienced by the cells. For MCF7 cells, a higher number of cells adhered to the nanosurface per unit area than those on the flat control surface (Figure 1I).

The circularity of the cells on the nanosurface got decreased, but it was not as significant as that of MDA-MB-231 cells (Figure 1J). This result demonstrated that polarization and elongation of MCF7 cells on the nanosurface were less significant than that of MDA-MB-231 cells. Regarding the projected area of cells, at high *T*_*tol*_, cells on the flat surface were larger than cells on the nanosurface (Figure 1K). Collectively, these results indicated that the MCF7 cells have a limited capability to change their morphology in response to the nanopattern compared to the highly metastatic cancer cells. MCF10A cells, on both surfaces, did not show a distinct difference in the number of adherent cells per unit area (Figure 1L). A less significant change in circularity than that of MDA-MB-231 cells and limited cell expansion on the nanosurfaces were noticeable, presumably due to the low susceptibility of the normal cell in response to the topographical cue (Figures 1M,N). Altogether, our results showed that cell adhesion and spreading are guided by integrin tension, and cells with high invasiveness have enhanced conformity to their architectural environment.

### Mapping of Integrin Tension Reveals Distinct Force Distribution in Human Breast Epithelial Cell Lines

**FIGURE 2:**
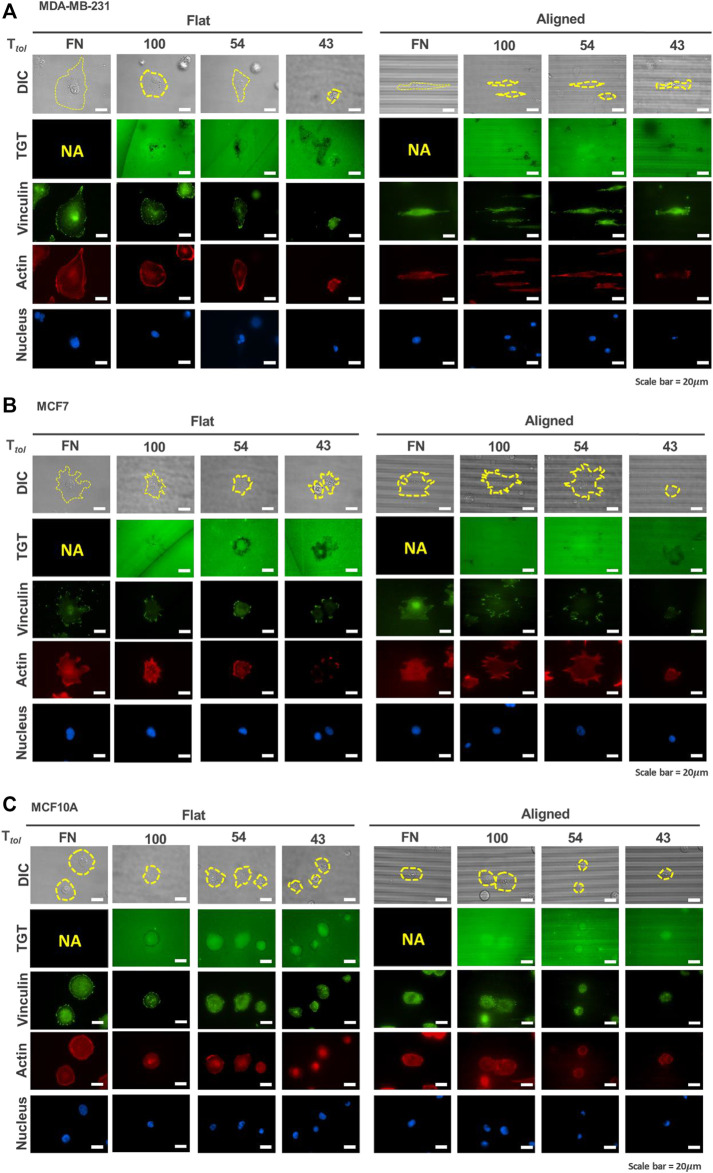
Mapping of integrin tension of human breast epithelial cells during adhesion on flat and nanopatterned surfaces. Cell adhesion to nanosurfaces with different *T*_*tol*_ (43, 54, and 100 pN) induced distinctive rupture patterns. Fluorescent signal loss in the TGT image channel occurs when strong integrin tensions greater than *T*_*tol*_ are applied to the integrin-ligand bonds due to the activity of invadopodia or force transmission through the focal adhesion indicated by using a focal adhesion marker, vinculin. MDA-MB-231 cells **(A)** caused TGT ruptures on the ventral surface of cells and at the cell periphery, while MCF7 **(B)** and MCF10A **(C)** formed rupture patterns mostly at their cell edges. Scale bar, 20 µm.

Using the topo-TGT assay, integrin tension on the ventral cell surface was mapped and analyzed to answer how the cell type and the topographical cue affect the generation of integrin tension. Since the TGT molecules were designed to be dissociated under higher forces than *T*_*tol*_, the cell-exerting integrin tension on the surface can be mapped by monitoring the loss of fluorescent signals (Figure 2). As reported before (Roein-Peikar et al., 2016), rupture patterns produced by cells are classified as follows: 1) a ring-like “edge rupture” (caused by strong forces on FA along the cell periphery with the activity of actomyosin) and 2) “entire rupture” (rupture formed in the entire cell contact area during early adhesion).

For MDA-MB-231 cells, on the flat surface, edge rupture was observed on the 54 pN TGT surface but not on the 100-pN surface (Figure 2A). An entire rupture was found on the 43 pN TGT surface. The staining of vinculin and actin indicated that FA was formed at the cell edge even on the 43 pN TGT surface. However, actin stress fibers, including dorsal stress fibers, transverse arcs, and ventral stress fibers, were not well developed on the 43 pN TGT surface. Analysis of the formation of focal adhesion and stress fibers manifested that integrin tension higher than 43 pN is needed for maturation of FA, and cell force mediated by mature focal adhesion at the cell edge was in a range of 54 and 100 pN. On the nanosurface, rupture patterns have changed. As the cells were highly elongated, firm adhesion complexes were manufactured at the cell tips, leading to augmented forces even enough to break the 100 pN DNA tether. In addition to the TGT ruptures, in all tested conditions, punctate fluorescent signal loss under cells (named “ventral rupture”) was observed. This might be caused by DNase recruited in invadopodia or invadopodia activity in the MDA-MB-231 cells (Pal et al., 2021; Kim et al., 2022). For the MCF7 cells, the ventral rupture was rarely found; instead, the edge rupture was dominant in all conditions (Figure 2B). On the flat surface, edge rupture was found even on the 100 pN TGT surface, while edge rupture was found only on the nanosurface with *T*_*tol*_ = 43 and 54 pN. Regarding stress fiber construction, this result suggested that mature FA formed on the flat surface transmitted strong tension higher than 100 pN, and the tension on the FA was alleviated using the nanopatterns due to pre-mature stress fiber formation. Last, for the MCF10A cells, similar to the results of the MCF7 cells, edge rupture was found in all surfaces except the 100-pN nanosurface, presumably due to the diminished tension of cells on the nanopatterns (Figure 2C). Altogether, these TGT results showed that unique rupture patterns found in each cell type indicate different mechano-sensitivities of cells to the nanotopographical cue, which influences the formation of FA and stress fibers and their alignments, eventually inducing an alteration in the tension applied on the integrin adhesion proteins.

### Reconstructed Layer-by-Layer Force Map Demonstrates the Magnitude of Spatial Integrin Tension on the Nanosurface

**FIGURE 3:**
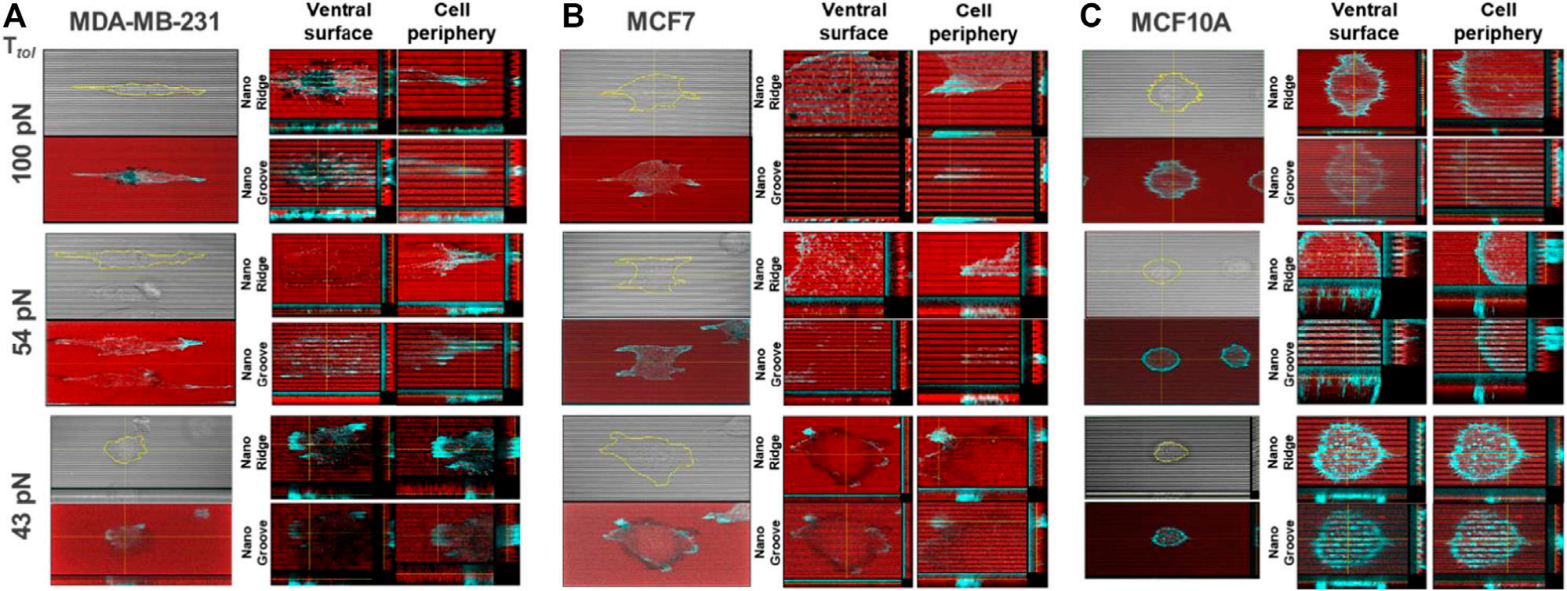
Reconstructed layer-by-layer force map of the three cell lines on the nanosurface. Each cell exerted different levels of integrin tensions on the nanoridges and the nanogrooves. The magnitude of spatial integrin tension on the nanosurface was analyzed with actin polymerization. The red color indicates the fluorescent signal from the immobilized-TGT, and the cyan color indicates F-actin. **(A)** MDA-MB-231 cells applied integrin tension higher than 100 pN to the nanoridges and nanogrooves at the ventral cell surface and the nanoridges at the cell tip. **(B)** MCF7 cells exerted integrin tensions in a range of 43–54 pN along the cell periphery. **(C)** MCF10A cells generated integrin tensions in a range of 54–100 pN along the cell periphery.

To further evaluate the magnitude of spatial integrin tension on the nanosurface at the subcellular level, we conducted the topo-TGT assay focusing on four regions: 1) nanoridges and 2) nanogrooves at the ventral surface of adherent cells and 3) nanoridges and 4) nanogrooves at the protrusion or tip of the adherent cells. Confocal images of the ruptures and F-actin filaments stained using the phalloidin in each position were taken and reconstructed for detailed layer-by-layer analysis. For MDA-MB-231 cells, cells filled the nanogrooves, and intracellular actin stress fibers were oriented along the nanopatterns, presumably because of the high compliance of the invasive cancer cells (Figure 3A). The ventral rupture was found on nanoridges and nanogrooves of the nanosurface with *T*_*tol*_ = 54 and 100 pN, indicating that invadopodia might directly contact the nanoridges and nanogrooves, and the consequent activity of DNase in invadopodia induced the rupture on the ventral cell surface. At the cell tips, the weak fluorescent signal loss was observed only on the 100 pN nanoridges. This implies a few 100 pN TGT molecules immobilized on the nanoridges were pulled away, whereas most TGT molecules on the nanogrooves remained intact. On the 54 pN surface, a stronger fluorescent signal loss was observed on the nanoridges and nanogrooves. It meant that the integrin tension on the nanoridges was >100 pN, and the integrin tension on the nanogrooves was 54–100 pN. On the 43 pN surfaces, a considerably reduced fluorescent signal was detected in the region underneath the cell due to the entire rupture on the nanoridges and nanogrooves. For MCF7 cells, unlike MDA-MB-231 cells, the cells perch on the nanoridges without forming actin stress fibers in the nanogrooves except for the tip of cell protrusions (Figure 3B). The ventral rupture was found sporadically on all tested TGT surfaces. At the cell periphery, the ruptures were discovered only on the surface with *T*_*tol*_ ≤ 54 pN, and the rupture patterns were distinguishable. On the 54 pN surface, small and weak ruptures were found at the tip of cell protrusions, while on the 43 pN surface, robust edge rupture was discovered along the cell edge. For MCF10A cells, similar to MDA-MB-231 cells, the whole cell body occupied the nanogrooves with aligned F-actin (Figure 3C). Ruptures on the nanoridges and nanogrooves were detected only at the cell periphery on the TGT surfaces with *T*_*tol*_ = 43 and 54 pN.

Moreover, to evaluate how well the topo-TGT assay reflects altered integrin tension by external biochemical cues, the topo-TGT assay with MDA-MB-231 cells treated with a drug for remodeling of cytoskeleton structures was performed. To modulate the cytoskeleton polymerization and subsequent contractility, MDA-MB-231 cells were preincubated with either a Rho activator (CN03) or a phosphoinositide 3-kinase (PI3K) inhibitor (LY294002) before seeding them on the nanosurface (Supplementary Figure S2A). Rho activator increases the level of guanosine triphosphate (GTP)-bound RhoA, provoking re-arrangement of the actin cytoskeleton (Li et al., 2021). Here, enhanced actin polymerization increased the obvious stiffness of the cells, making them less elongated on the 54 pN TGT nanosurface. Thicker and more robust edge ruptures along round-cell periphery suggested that integrin tension on the nanoridge got increased, higher than 54 pN, presumably as an outcome of the cortical actin development. Surprisingly, with the PI3K inhibitor, actin fibers penetrating nanogrooves in the region of the ventral cell surface disappeared, and the TGT ruptures, except the ventral rupture, were considerably weaker or diminished. It is predicted that since PI3K is closely linked to actin polymerization (Jiménez et al., 2000), inhibition of the PI3K inhibitor might result in interrupted actin polymerization at the ventral surface and consequently decreased contractility and integrin tension at the contact surface. To summarize, the topo-TGT assay manifests altered cells’ morphology, cytoarchitecture, and adhesion strength inside nanostructures together.

## Discussion

**FIGURE 4:**
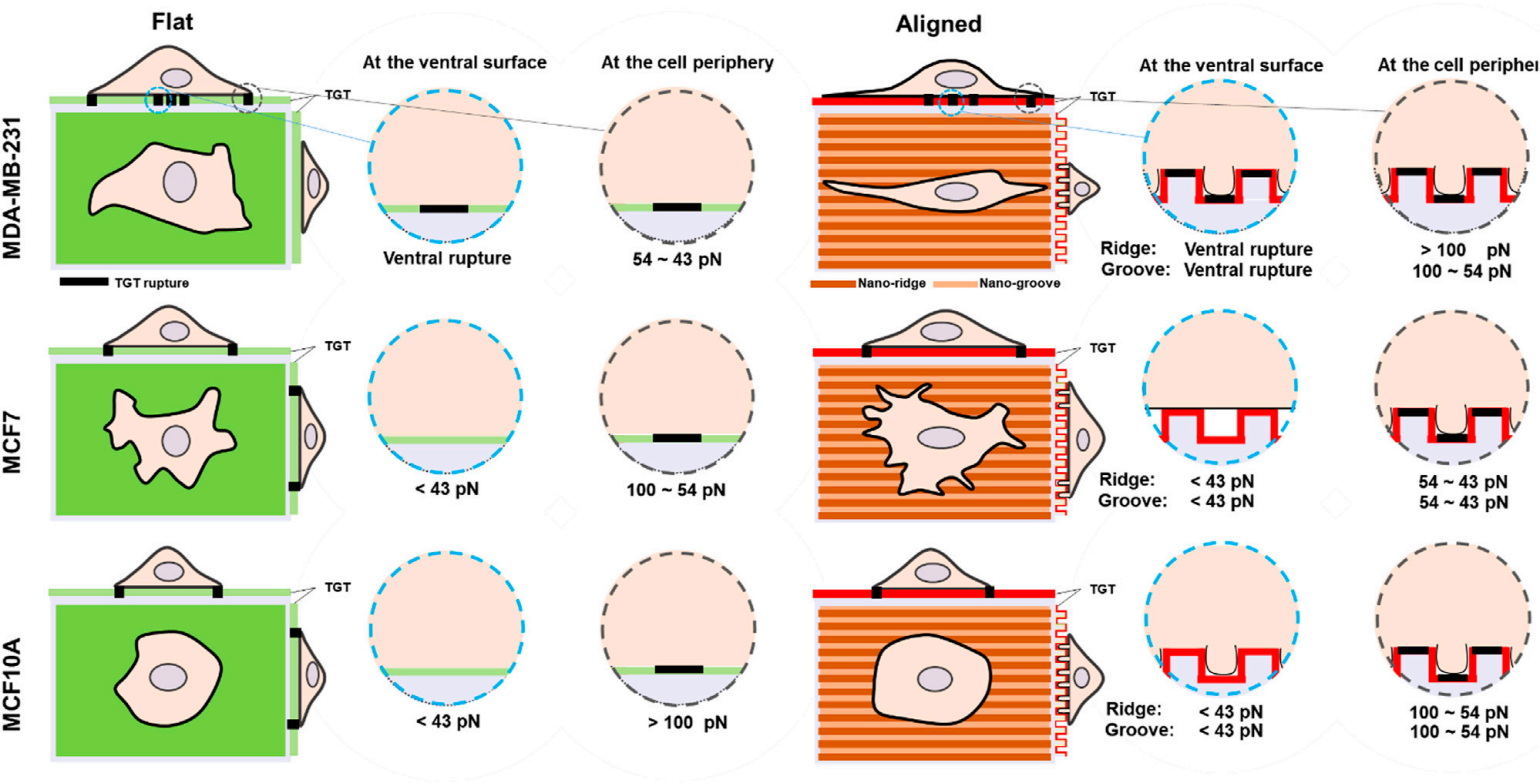
Schematic comparison of molecular integrin tension exerted by three different human breast epithelial cells during adhesion and spreading on flat and nanosurfaces.

Previous studies have shown that topographical cues displayed in the ECM significantly influence cell morphology and adhesion (Kim et al., 2012). Also, cell responses to the topographies are highly cell type-dependent. In this study, we compared morphology and adhesion strength on nanosized topography using three cell lines with different invasiveness and analyzed the level of cell force on integrin-ligand bonds with nanoscale spatial resolution and single-molecule force precision. The most notable differences across the tested cell lines were the capability of compliance to the nanotopographies, the magnitude of the spatial integrin tension, and the existence of ventral rupture summarized in Figure 4. The highly invasive breast cancer cells MDA-MB-231 indicated dramatic morphological changes in response to the grooved nanofeatures; 1) elongation along the orientation of the patterns and 2) deformation enough to fill the nanogaps. These changes in the cell shape accompanied changes in the physical force generation at the cell periphery. The magnitude of integrin tension increased to 54–100 pN due to the development of directed ventral and dorsal stress fibers in the cell tips. On the nanosurface, non-invasive cancer cells (MCF7) showed less elongated morphology and adhesion on the nanoridges without penetration into the nanogrooves, owing to the higher plasticity. Only protrusions filled the nanogap with weakened integrin tension in a range of 43–54 pN MCF10A cells on the nanosurface exhibited significantly reduced cell area with penetration into the nanogrooves, and their integrin tension applied to the cell periphery got lowered from >100 pN to 54–100 pN but still greater than that of MCF7 cells. Taken together, our results illustrated that similar to cell adhesion on the flat surface, cell adhesion to the nanosurfaces is activated with integrins that experience tension greater than the universal integrin tension of 40 pN (Wang and Ha, 2013). However, the forces transmitted through the integrin-ligand bond in mature FA vary depending on the cell type or external biochemical cues. In addition to ruptures at the edge or tip, most noticeably, metastatic cancer cells and a small population of non-invasive cancer cells produced ventral rupture because of the activity of invadopodia. This suggests that the invadopodia form and operate without being affected by the topographical cues, and the existence of ventral rupture can be used as a metastatic cancer marker.

In this brief report, using the topo-TGT assay, we successfully measured the molecular integrin tension beneath the cells adhered to the nanotopography mimicking individual collagen nanofibers existing in an ECM layer that cells may encounter or experience (Ray et al., 2017). Thus, it allowed us to understand how substrate topography influences cell morphology and adhesion depending on the cell type, of note, how metastatic cancer cells are directed toward the CG by changing their cytoskeleton dynamics and force generation. Our study was conducted with a defined nanofeature with a uniform period (800 nm ridge × 800 nm groove × 600 nm height); however, in an *in vivo* environment, nanoarchitecture with various sizes and periods exist, and they may have a different impact on cell morphology and adhesion. Previously, it was reported that cell penetration into the groove is limited when the grooves are too narrow, and cells lose extra contact area caused by the topography when the grooves are too wide or the surface is flat or rough (Kim et al., 2010; Anselmi et al., 2021). In addition, we observed that adhesion of MDA-MB-231 cells to the nanosurface with the low TGT density was dramatically reduced, indicating the ligand density and spacing may have a profound impact on cell adhesion (Supplementary Figure S3). For these reasons, further comprehensive studies need to be undertaken with different nanopatterns with different geometries, dimensions, ligand densities, and different ligand interactions to better elucidate the biological mechanism during CG. Moreover, CG ultimately leads to cell migration. Although we focused only on cancer cell adhesion in this study, this can be expanded to eventually understand the mechanisms of cancer invasion and metastasis across the nanotopographical surfaces. Therefore, spatio-temporal analysis of the topographical cue-induced integrin tension during cell migration should be examined to understand the mechanisms underlying cancer metastasis in future studies.

In conclusion, this brief research report introduces a novel platform designed to quantify tension experienced by integrin during adhesion to the nanotopography and the resulting morphological and biophysical changes in cancer cell lines. The biophysical information will provide us a valuable insight into the relationship between the cell-substrate interaction and cellular processes, ultimately used to answer unknown mechanobiological mechanisms of cancer progression and metastasis.

## Data Availability

The original contributions presented in the study are included in the article/Supplementary Material, further inquiries can be directed to the corresponding author.
