# Supplementary material for: Characterization of Integrin Molecular Tension of Human Breast Cancer Cells on Anisotropic Nanopatterns
Source: Front Mol Biosci. 2022 Jun 9;9:825970. doi: 10.3389/fmolb.2022.825970 (PMC9218603; doi:10.3389/fmolb.2022.825970)
Supplement: Supplementary file 1 [file DataSheet1.docx]

# Supplementary Material

Characterization of integrin molecular tension of human breast cancer cells on anisotropic nanopatterns

Kyung Ah Kim^1^, Srivithya Vellampatti^1^, and Byoung Choul Kim^1*^

^1^Department of Nano-bioengineering, Incheon National University, Incheon, Republic of Korea


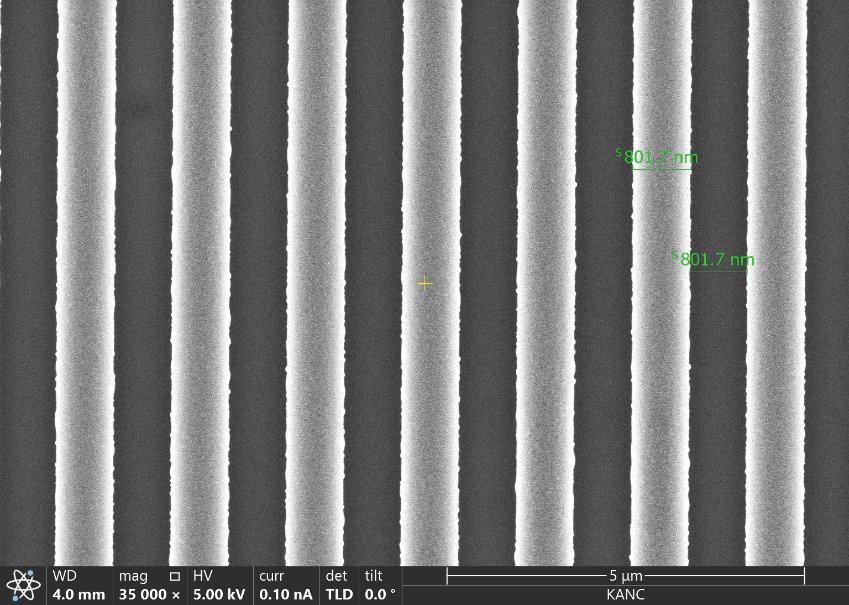


**Supplementary Figure S1.** A scanning electron microscope (SEM) image of the nanosurface with the aligned anisotropic pattern. Measured width and ridge were approximately 800 nm. Scale bar = 5 µm.

**
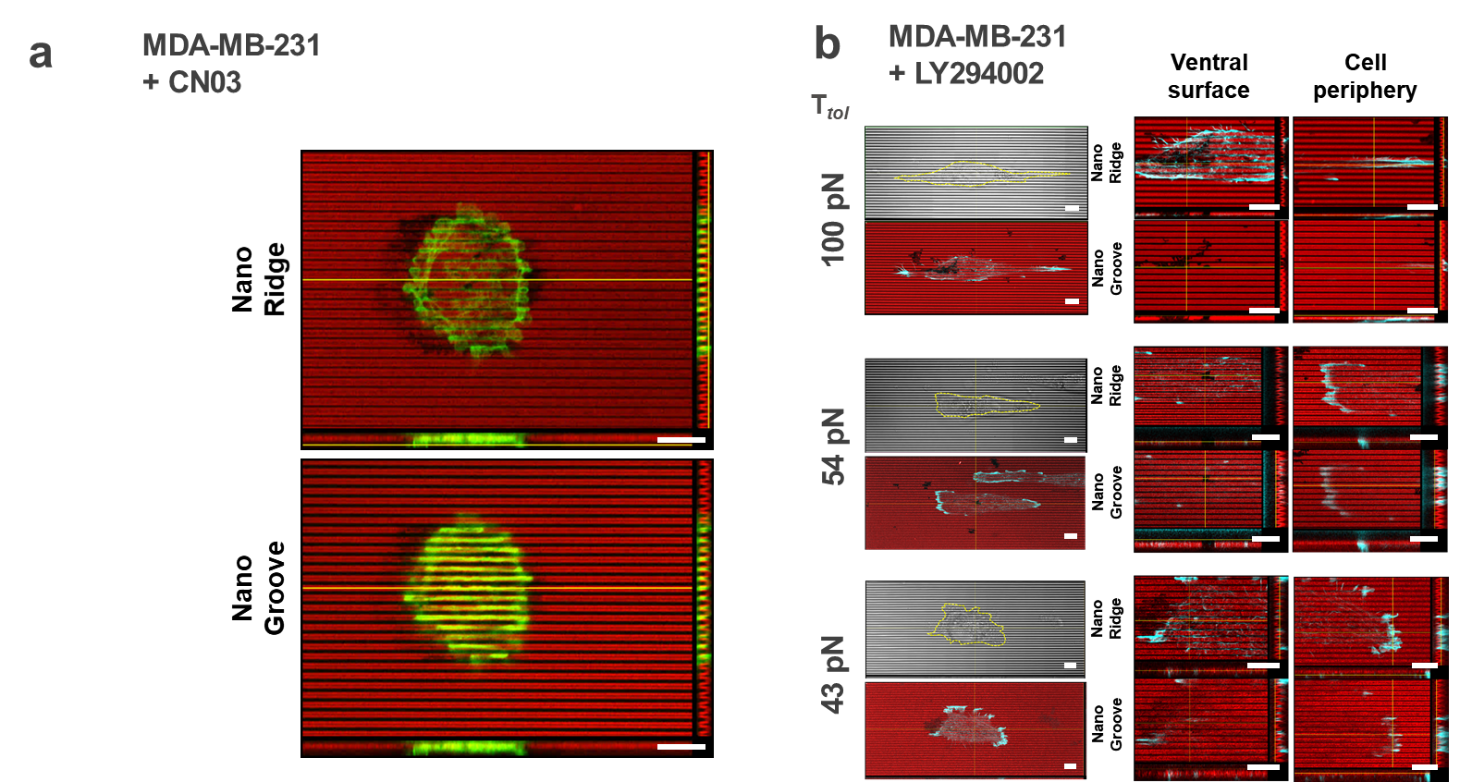
**

**Supplementary Figure S2. Topo-TGT assay with MDA-MB-231 cells treated a Rho activator or a PI3K inhibitor.** TGT ruptures formed by MDA-MB-231 cells treated with either CN03 (Rho activator) or LY294002 (PI3K inhibitor) on the 54 pN TGT nanosuface were monitored. Red color indicates the immobilized TGT and green color or cyan color indicates F-actin. (a) MDA-MB-231 cells treated with CN03 maintained the round shape instead of being elongated and induced thicker and robust edge rupture at cell periphery. (b) Rupture at the cell tips caused by the MDA-MB-231 cells treated with LY294002 became weaker or disappeared. However, the ventral rupture was still observed.


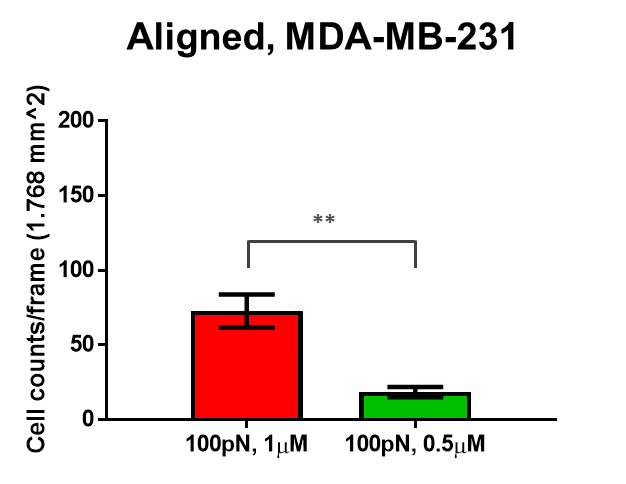


**Supplementary Figure S3. MDA-MB-231 cell adhesion on the nanosurface coated with different concentrations of 100 pN TGT molecules.** The number of adherent cells on 100 pN nanosurfaces with different TGT concentration (0.5 µM vs 1 µM). All experiments were repeated three times.
